# Supplementary material for: Evaluation of the Preclinical Efficacy of Lurbinectedin in Malignant Pleural Mesothelioma
Source: Cancers (Basel). 2021 May 12;13(10):2332. doi: 10.3390/cancers13102332 (PMC8151304; doi:10.3390/cancers13102332)
Supplement: Supplementary file 1 [file cancers-13-02332-s001.zip › cancers-1210822-SI.pdf]

Supplementary materials

Evaluation of the preclinical efficacy of lurbinectedin in malignant pleural mesothelioma

Supplementary Figure S1

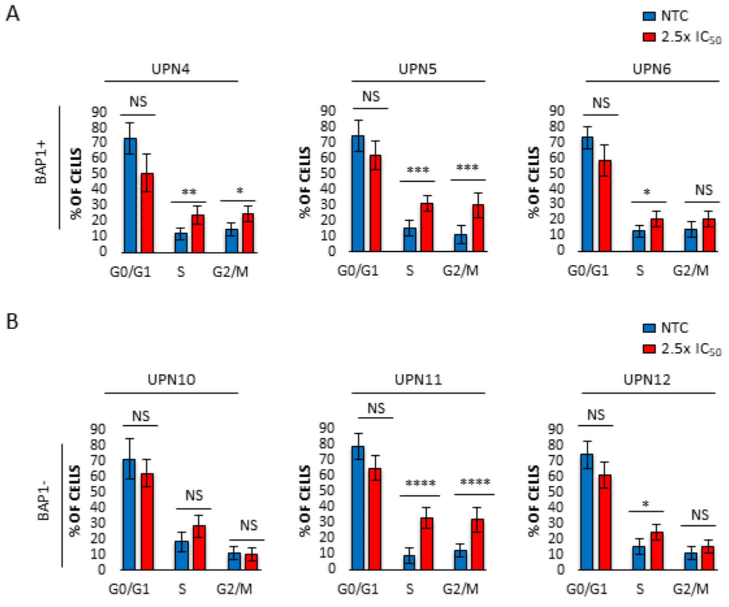

Supplementary Figure S1. Lurbinectedin effects on cell cycle distribution. (**A**,**B**) Histograms showing cell number percentage in each cell cycle phase (G0/G1, S and G2/M) of the indicated MPM cell lines, treated or not with lurbinectedin (2.5-fold the IC_{50}) for 24h. Data are expressed as means ± SEM; $^{NS}P>0.05$; * $p < 0.05$; ** $p < 0.01$; *** $p < 0.001$; **** $p < 0.0001$

Supplementary Figure S2

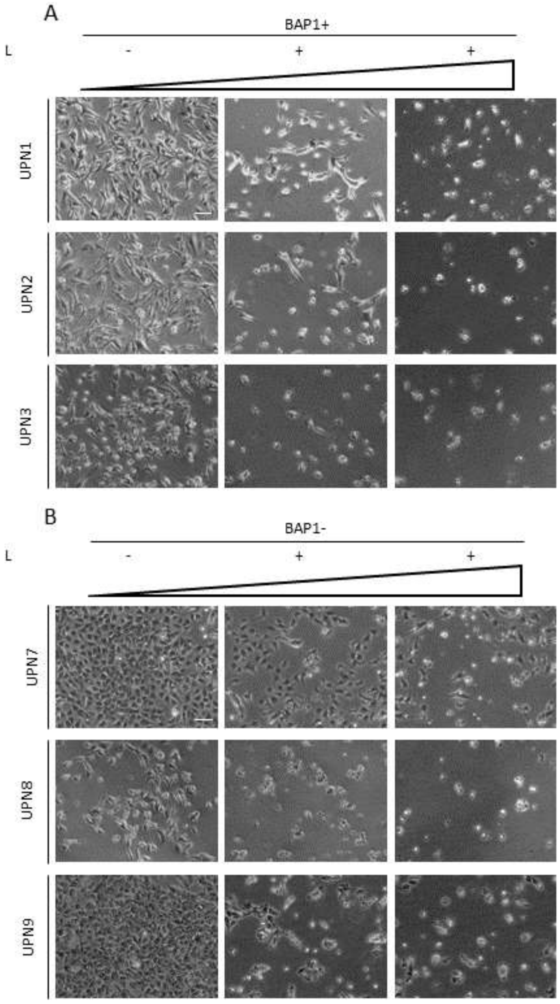

Supplementary Figure S2. Lurbinectedin strongly impairs cell viability in MPM cell lines. (**A**,**B**) Representative microscopic images of the indicated MPM cell lines treated or not with two different lurbinectedin (L) concentrations (2.5-fold and 5-fold the IC_{50}) for 72h (scale bar = 100μm).

Table S1. Histological features of the original tumors, clinical features and treatments subsequently received of the corresponding patients

UPN	Histotype	BAP1	Gender	Age	Asbestos exposure	Surgery	First-line treatment	Second-line treatment	OS (months)
1	epithelioid	POS	M	78	Possible	No	Palliative	No	6
2	epithelioid	POS	M	69	Yes	Yes*	No	No	12
3	epithelioid	POS	M	70	Yes	Yes*	No	No	3
4	epithelioid	POS	M	79	Possible	No	Carbo+Pem	Gem	16
5	epithelioid	POS	M	68	Yes	No	Carbo+Pem	Pem	9
6	sarcomatoid	POS	M	69	Yes	No	Carbo+Pem	Trabectedin	10
7	sarcomatoid	NEG	M	77	Yes	No	No	No	3
8	epithelioid	NEG	M	53	Yes	Yes	No	No	13
9	epithelioid	NEG	M	74	NR	No	Carbo+Pem	No	13
10	epithelioid	NEG	F	46	Yes	No	Carbo+Pem	Trabectedin	12
11	biphasic	NEG	M	60	Yes	No	Carbo+Pem	Gem+Vin	23
12	sarcomatoid	NEG	F	80	Yes	No	Carbo+Pem	Trabectedin	5

POS: positive; NEG: negative; M: male; F: female; NR: not reported; Yes*: Surgery + adjuvant platinum-based chemotherapy; n.a.: not availabe; Carbo: carboplatin; Pem: pemetrexed; Gem: gemcitabine; Vin: vinorelbine; OS: overall survival.
