# Supplementary material for: Evaluation of the Preclinical Efficacy of Lurbinectedin in Malignant Pleural Mesothelioma
Source: Cancers (Basel). 2021 May 12;13(10):2332. doi: 10.3390/cancers13102332 (PMC8151304; doi:10.3390/cancers13102332)

Whole blots Figure 1D (X-ray film development)

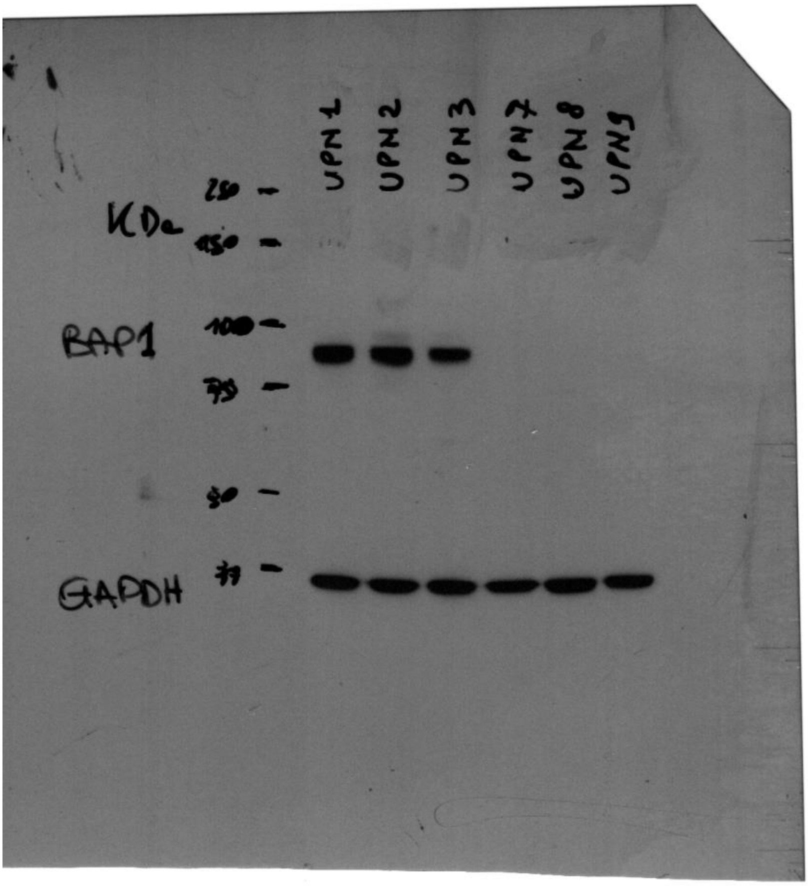

Densitometry analysis Figure 1D
(ImageJ software:https://imagej.nih.gov/ij/)

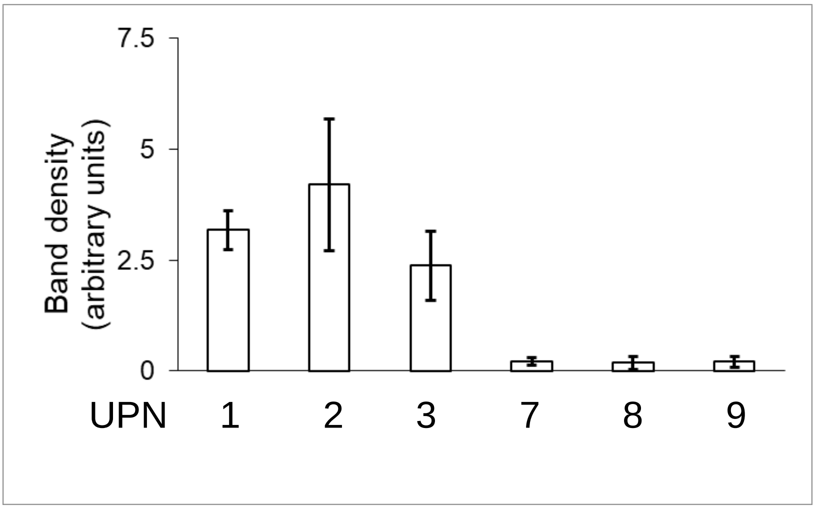

Whole blots Figure 5C-D (X-ray film development): UPN1, UPN7

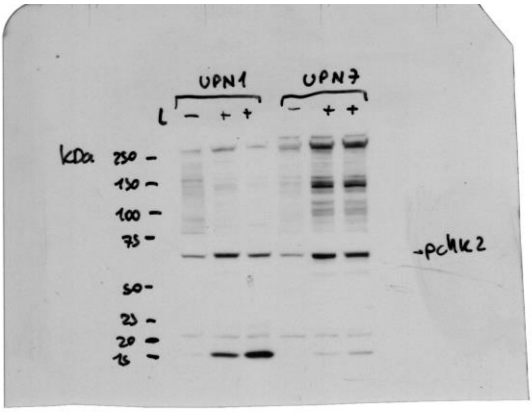

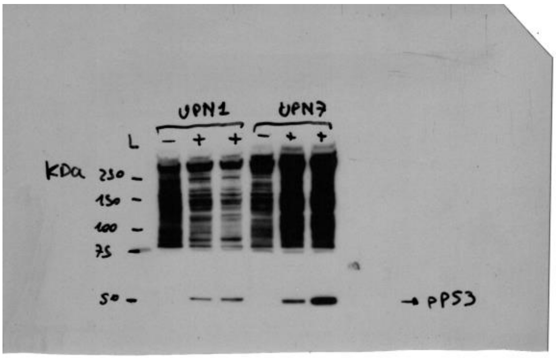

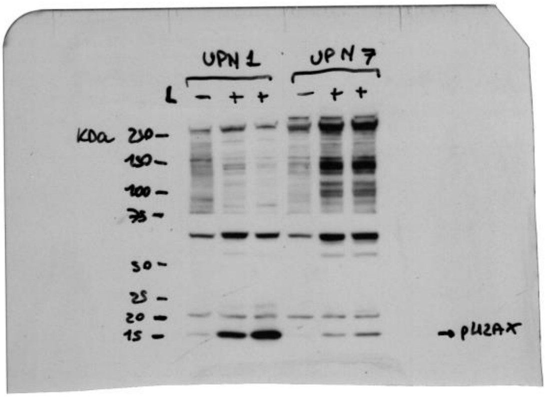

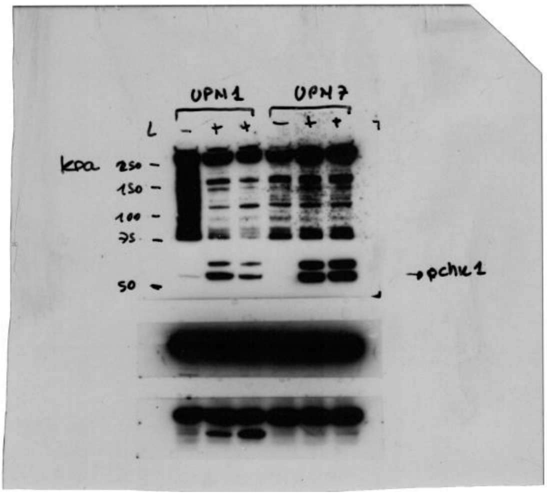

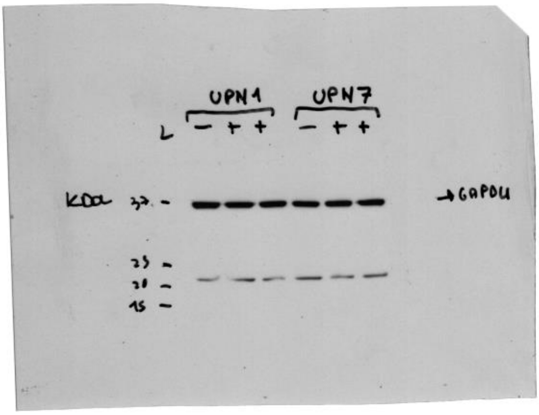

Whole blots Figure 5C-D (X-ray film development): UPN2, UPN8

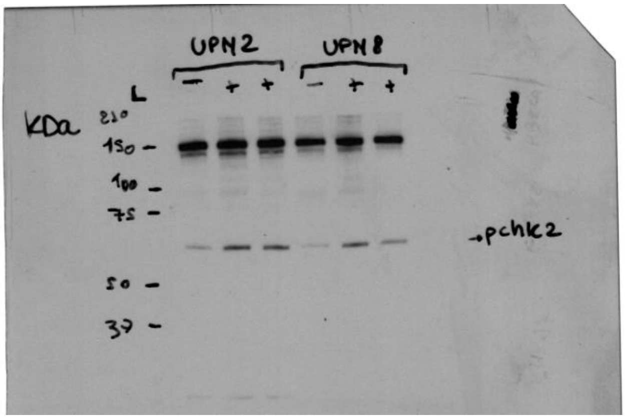

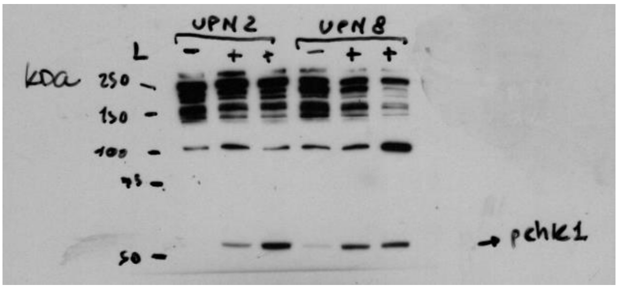

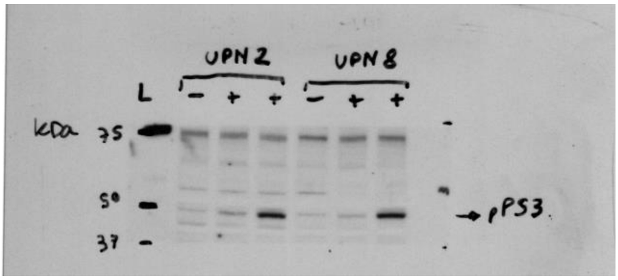

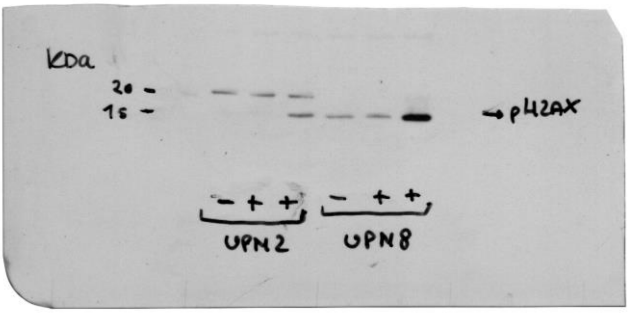

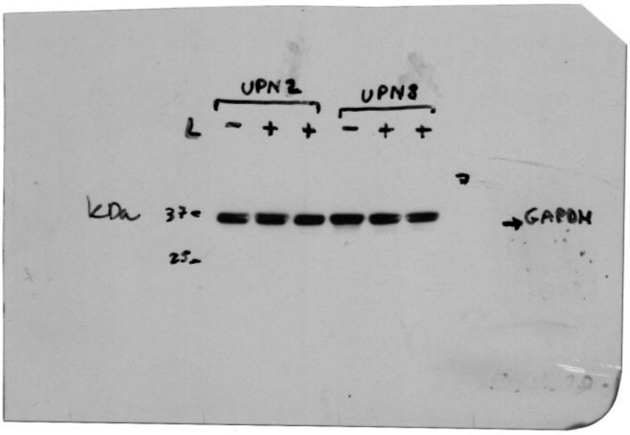

Whole blots Figure 5C-D (X-ray film development): UPN3, UPN9

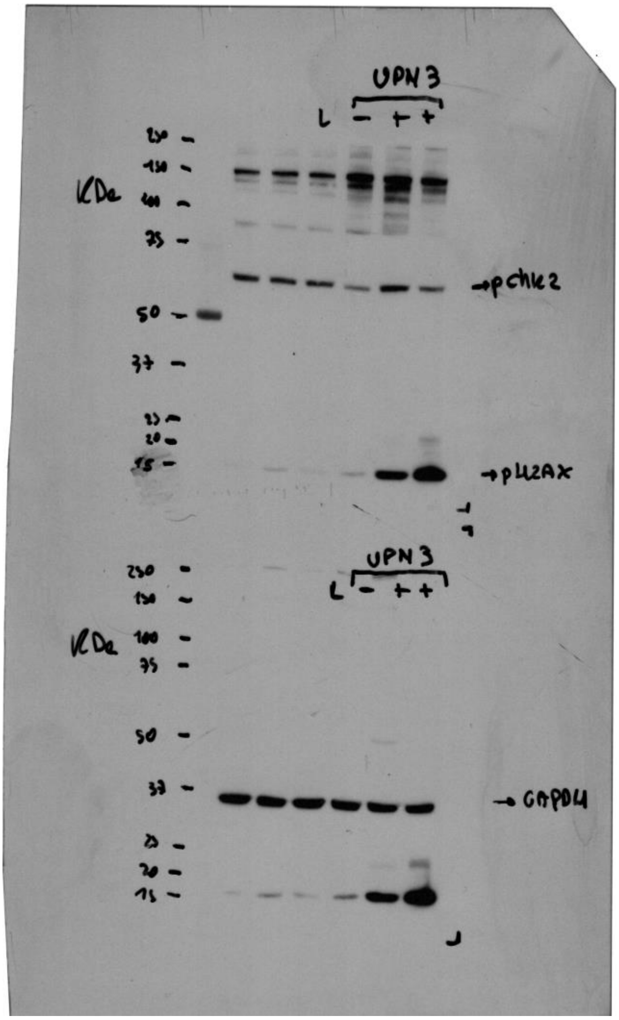

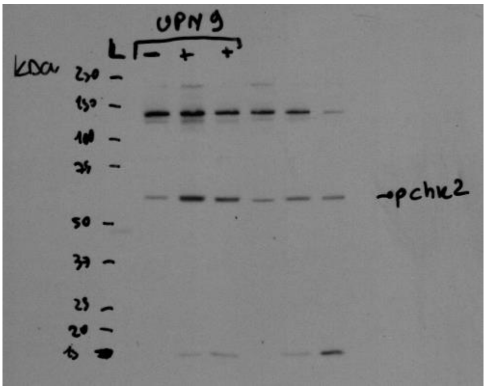

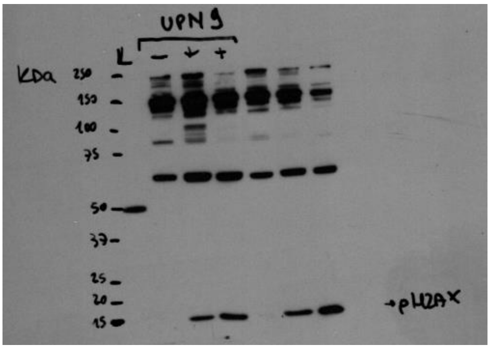

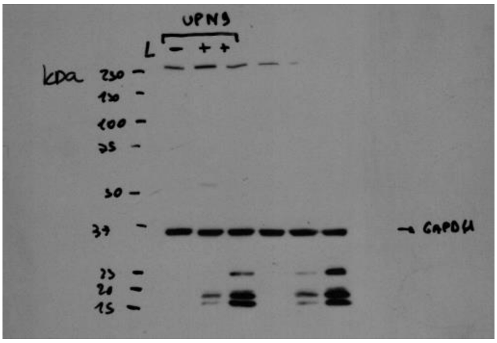

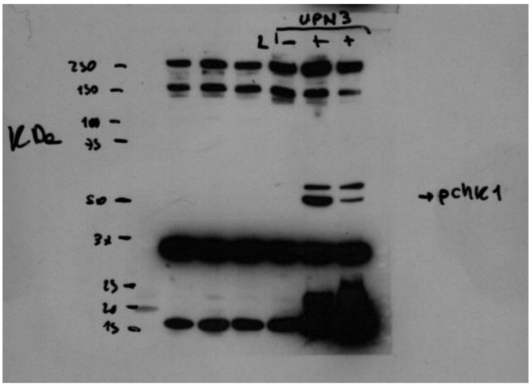

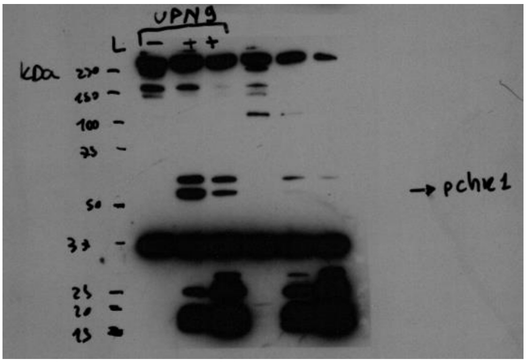

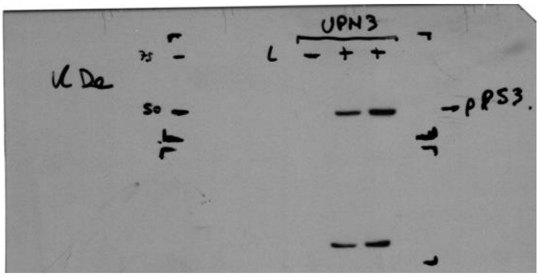

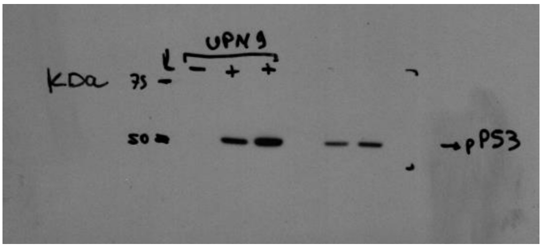

Densitometry analysis Figure 5C-D
(ImageJ software:https://imagej.nih.gov/ij/)

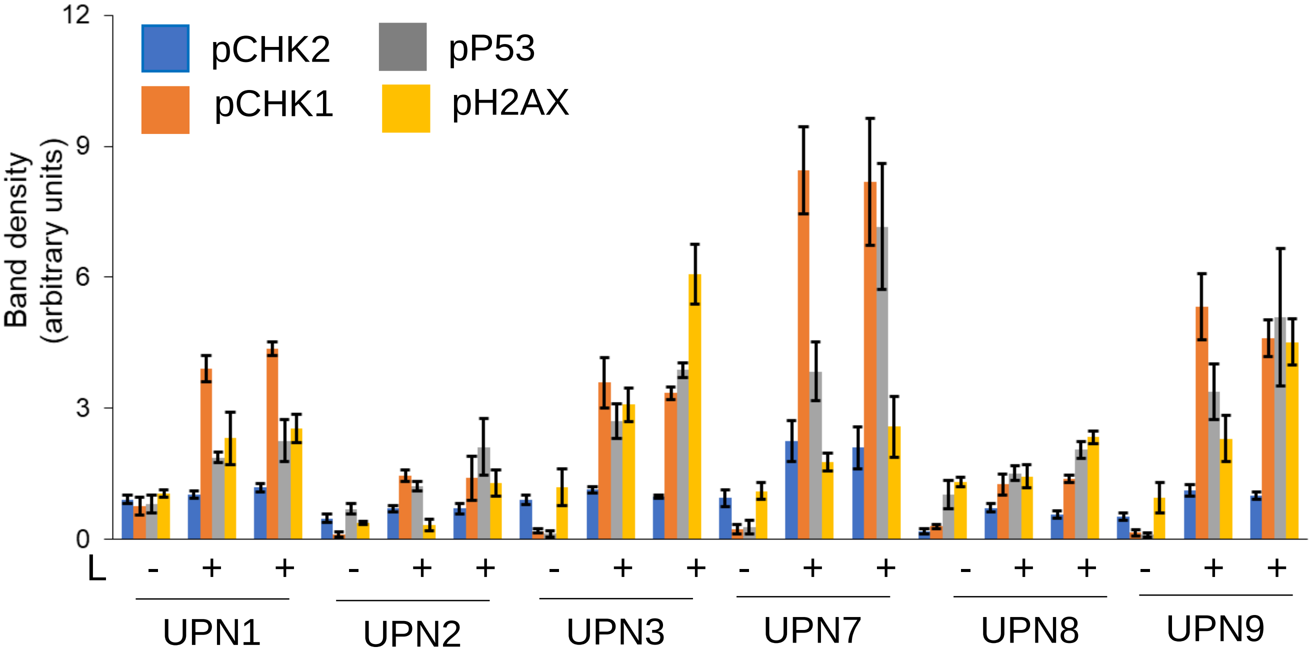

Whole blots Figure 6C-D:digital camera development (ChemiDoc, Bio-Rad)

UPN1

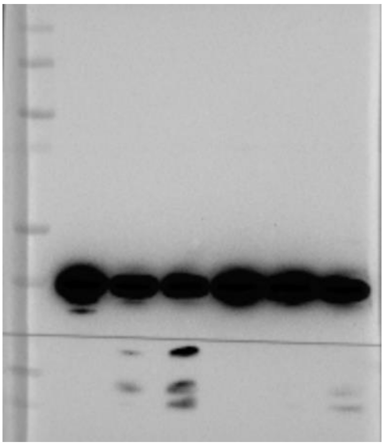

cl. casp 3

UPN7

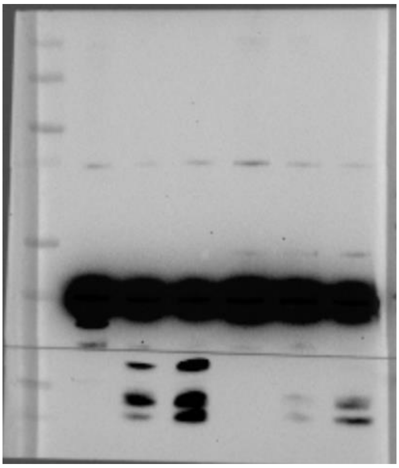

cl. casp 3

UPN1 UPN7

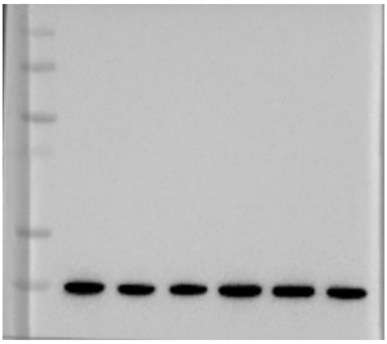

GAPDH

Whole blots Figure 6C-D:digital camera development (ChemiDoc, Bio-Rad)

UPN2 UPN8

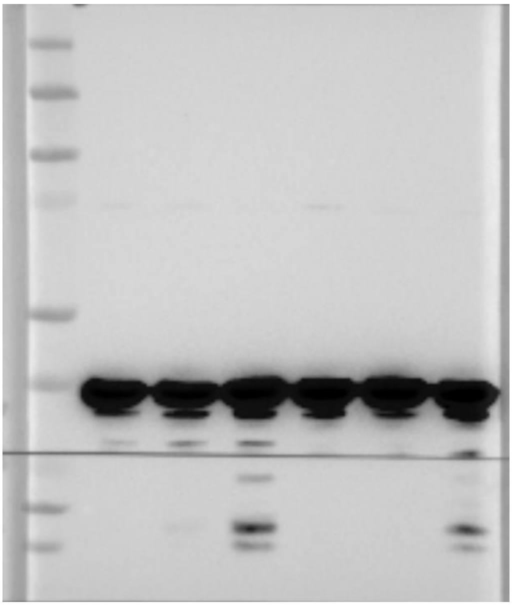

cl. casp 3

UPN2 UPN8

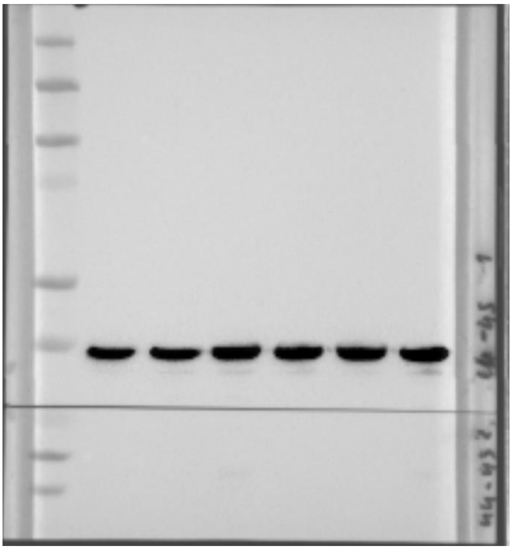

GAPDH

Whole blots Figure 6C-D:digital camera development (ChemiDoc, Bio-Rad)

UPN3 UPN9

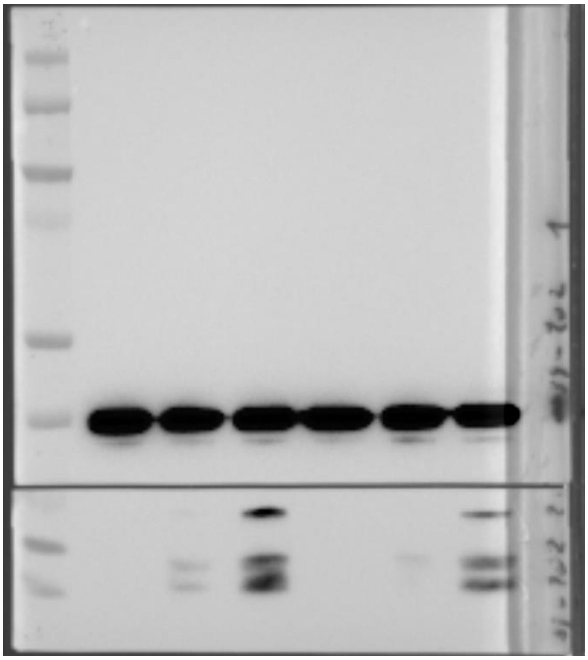

cl. casp 3

UPN3 UPN9

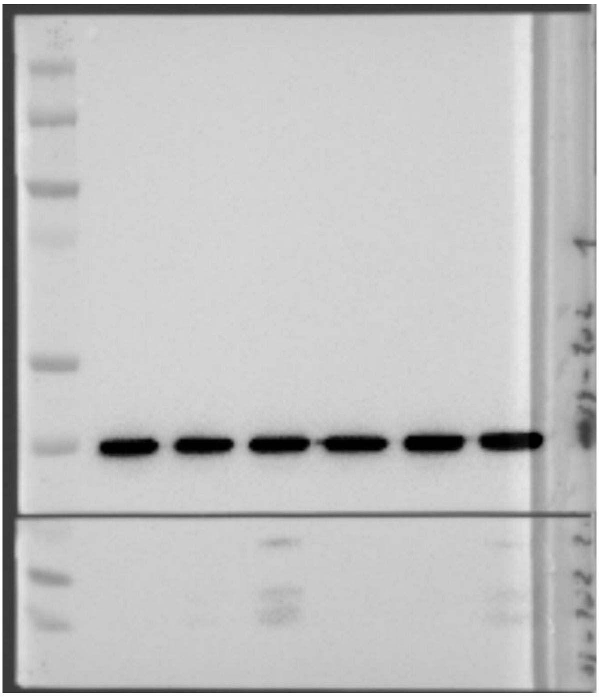

GAPDH

Densitometry analysis Figure 6C-D
(ImageJ software:https://imagej.nih.gov/ij/)

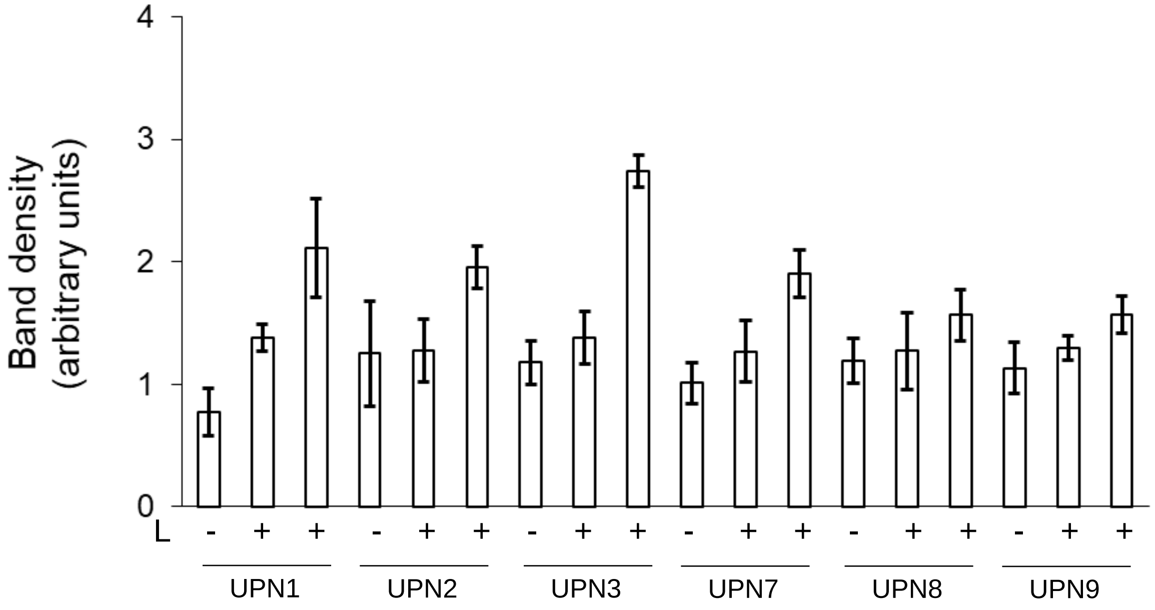

Supplement: Supplementary file 1 [file cancers-13-02332-s001.zip › Whole blots and densitometry analysis.pdf]
