# Supplementary figures and images for: Evaluation of the Preclinical Efficacy of Lurbinectedin in Malignant Pleural Mesothelioma
Source: Cancers (Basel). 2021 May 12;13(10):2332. doi: 10.3390/cancers13102332 (PMC8151304; doi:10.3390/cancers13102332)

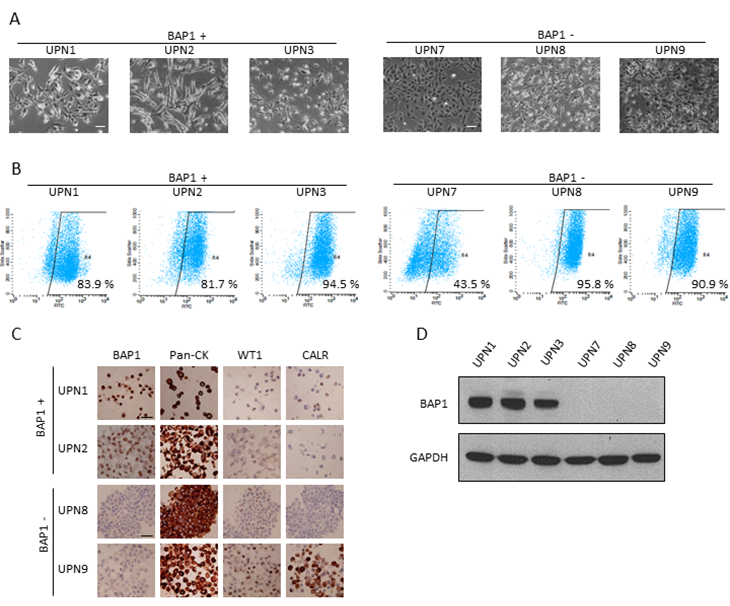

Supplement: Supplementary file 1 [file cancers-13-02332-s001.zip › Anobile Figures/Figure 1.tif]

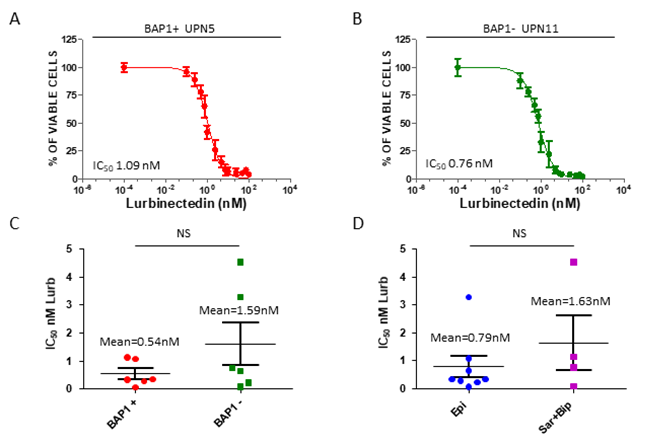

Supplement: Supplementary file 1 [file cancers-13-02332-s001.zip › Anobile Figures/Figure 2.tif]

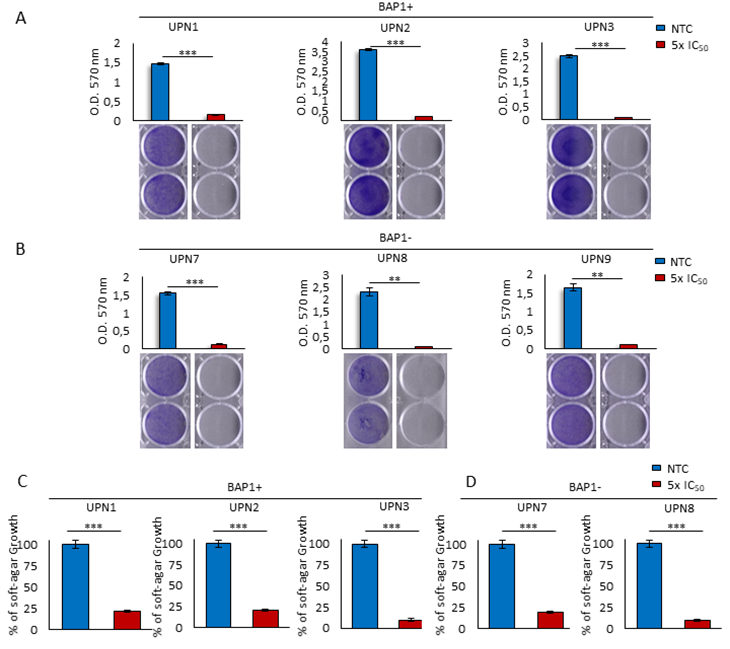

Supplement: Supplementary file 1 [file cancers-13-02332-s001.zip › Anobile Figures/Figure 3.tif]

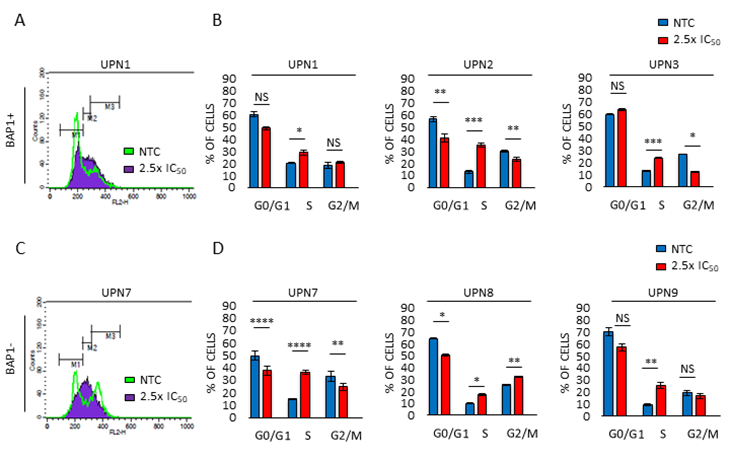

Supplement: Supplementary file 1 [file cancers-13-02332-s001.zip › Anobile Figures/Figure 4.tif]

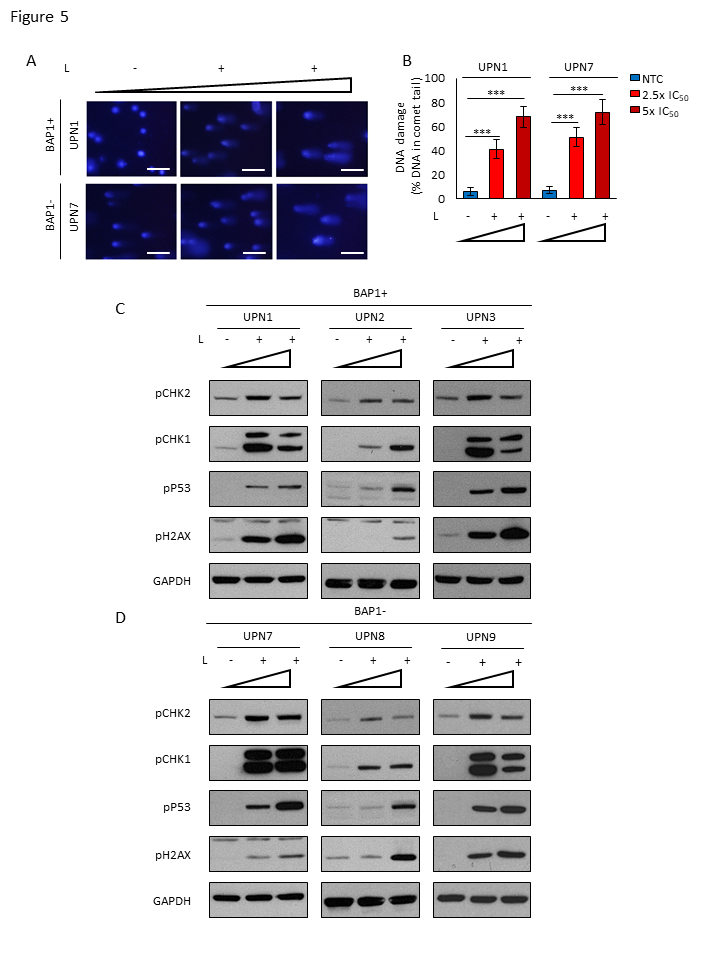

Supplement: Supplementary file 1 [file cancers-13-02332-s001.zip › Anobile Figures/Figure 5.tif]

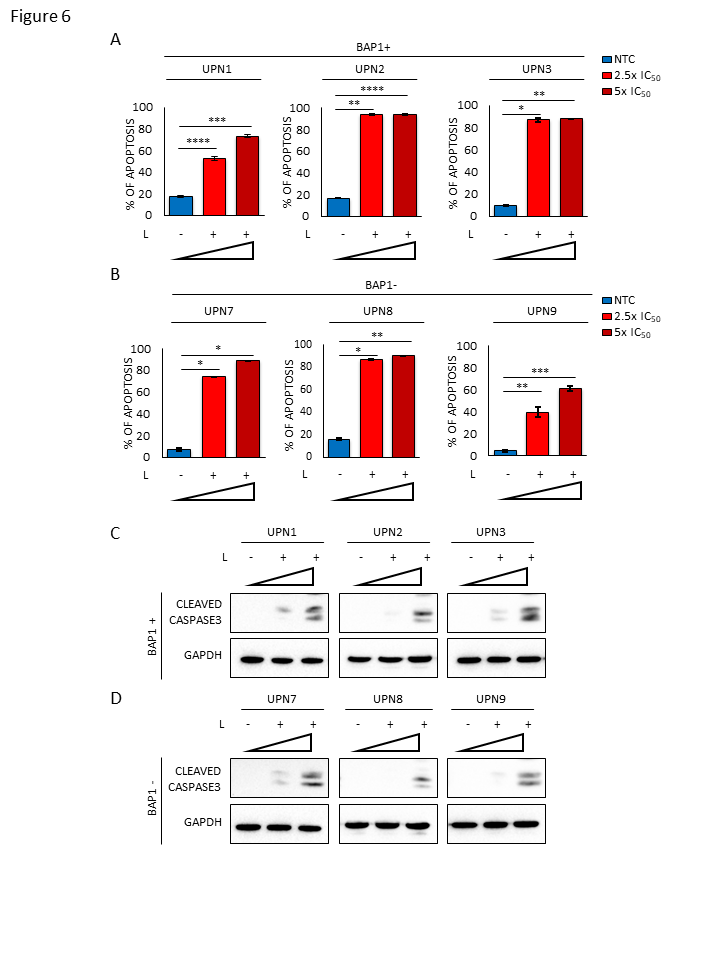

Supplement: Supplementary file 1 [file cancers-13-02332-s001.zip › Anobile Figures/Figure 6.tif]

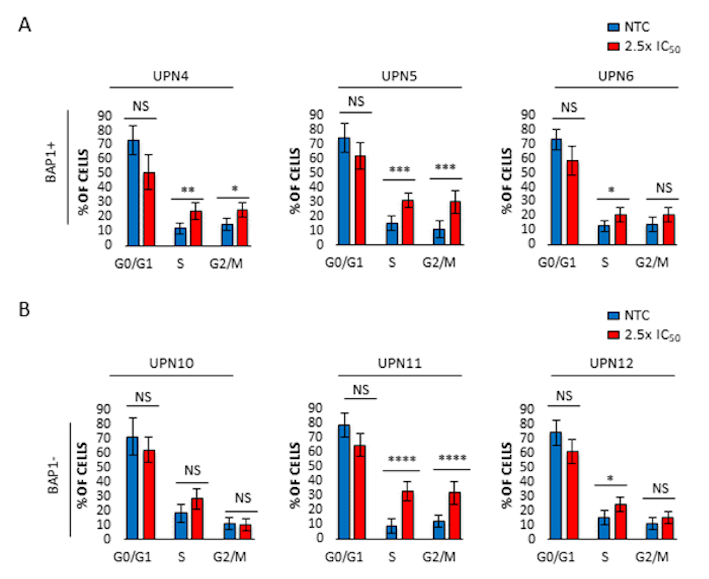

Supplement: Supplementary file 1 [file cancers-13-02332-s001.zip › Anobile Figures/Figure S1.tif]

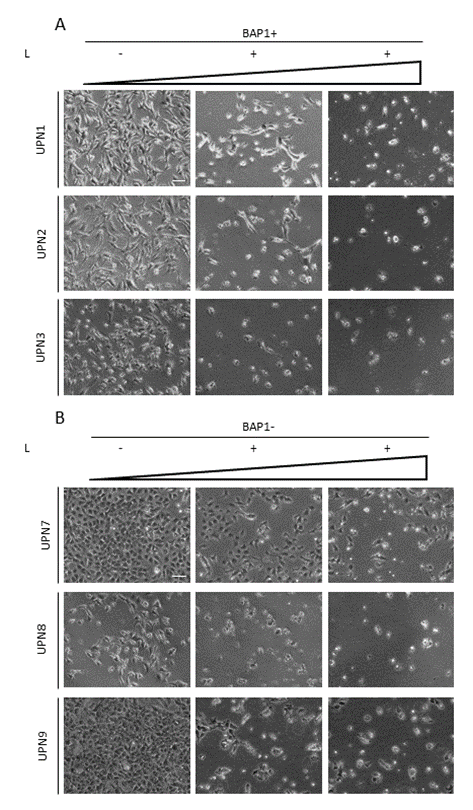

Supplement: Supplementary file 1 [file cancers-13-02332-s001.zip › Anobile Figures/Figure S2.tif]
